# Guest Edited Collection: Quantitative and computational techniques in optical coherence tomography

**DOI:** 10.1038/s41598-022-15424-y

**Published:** 2022-07-12

**Authors:** Peter Munro

**Affiliations:** University College London, London, UK

**Keywords:** Imaging and sensing, Three-dimensional imaging

## Abstract

Optical coherence tomography (OCT) is a three-dimensional optical imaging technique, frequently (but not exclusively) used for retinal imaging, that was first reported in the early 1990s. Since this time the technological development of OCT has been strongly influenced by its potential as a medical imaging technique. The first clinical prototype for use in ophthalmology was completed in 1994, paving the way for the first commercially available ophthalmic OCT system to be released to the market in 1996. Since then, OCT has become a mainstay of ophthalmology. OCT is also widely used in research, in an array of biomedical applications, and increasingly in industrial settings. Although there is still much activity in advancing OCT technology, there has been an increased emphasis in applying OCT to translational research. One direction of this research is in the development of quantitative and computational techniques to aid in the retrieval of clinically useful information from OCT images. This Collection brings together original research articles, which exploit realistic mathematical models of OCT image formation and machine learning approaches to obtain insight not otherwise available from raw OCT images. This includes research for measuring clinically relevant parameters such as retinal nerve fibre layer thickness, fractional flow reserve, and corneal biomechanics, and for performing feature identification and image process tasks.

Quantitative techniques seek to extract absolute measurements of sample-specific parameters which should be independent of the OCT system used to acquire the image. An example of such a technique is parametric imaging, where the optical scattering coefficient of tissue is measured throughout a sample’s volume^1^. This technique should ideally result in a three-dimensional distribution of scattering coefficient which is independent of the employed OCT system, and which may help in discerning features of clinical interest. Blood flow measurement is another example of an OCT-derived quantitative measurement, which provides some insight into tissue function^2^. Flow imaging harnesses OCT’s high displacement sensitivity, based on phase-sensitive detection, to make clinically relevant measurements of blood flow in tissues. Other examples of quantitative techniques based on OCT data include elastography^3^ and birefringence imaging^4^, which provides insight into the stiffness of tissues, and the organization of fibrillar structures, respectively.

Computational techniques which make use of simulations based on the physics of image formation in OCT have been developed to aid image interpretation. For example, computational adaptive optics^5^ aims to use computational methods to overcome sample- and system-induced aberration. Full-wave modelling techniques have also been developed^6^, which allow for highly realistic simulation of image formation in OCT, providing a potential solution to the problem of inverse scattering in OCT data, and allowing for an improvement of image interpretation.

Quantitative and computational techniques can thus be considered to perform one or more of the following tasks: quantitative parameter retrieval, image enhancement, and/or classification. Recently, an explosion in the number of machine learning approaches has been reported, which supplements algorithmic approaches for performing these tasks, and facilitates more accurate diagnoses from OCT data. Algorithmic approaches make use of physics-based or empirical models whereas machine learning approaches are largely unaware of such models.

Deep learning has been shown to exceed the performance of experts in making referral recommendations for a range of retinal diseases^7^. This is just one example from a rapidly developing field. This Guest Edited Collection incorporates a number of approaches, including studies which employ machine learning to perform the tasks of quantitative parameter retrieval, image enhancement and/or classification.

For example, Mariottoni et al.^8^ report a deep learning approach for measuring retinal nerve fibre layer (RNFL) thickness, the assessment of which is known to be important for the early diagnosis of glaucoma. Cha et al.^9^ report on a learned approach to measuring fractional flow reserve (FFR) from OCT data. The measurement of FFR is important for diagnosing myocardial ischemia, and the ability to make this measurement from OCT data, which also provides anatomical information, is of high clinical value.

Machine learning approaches for image enhancement and feature identification are also included in the Collection, such as that by Apostolopoulos et al.^10^, who report a method for postprocessing OCT B-scans that enhances image quality. The Collection also includes learned approaches to feature identification and classification, such as Lu et al., who characterize a method^11^ for analyzing intravascular optical coherence tomography images. Some papers included in the Collection describe multiple tasks, such as the work of Tsuji et al.^12^, who demonstrate a deep learning approach to segmentation of the choroid (a classification task) and for measuring the volume of the choroid (a parameter quantification task).

This Collection also includes papers which use algorithmic techniques, to perform the tasks mentioned above. For example, Yamanari et al.^13^ show that metrics related to the depolarization of light can be used to quantify melanin concentration in the eye, and Pitre et al.^14^ report a model of corneal biomechanics, validated by experiment, which may pave the way for acquiring clinically relevant measurements of corneal stiffness. The Collection also includes an algorithmic approach to image enhancement reported by Zhao et al.^15^, who make use of a model of image formation to correctly perform angular compounding, over large fields of view, to perform speckle reduction.

All of the papers included in this Guest Edited Collection aim to extract, or aid in the extraction of, clinically relevant information from OCT images. These approaches aim to deliver automatic, objective diagnoses of diseases, which will be essential given the rapidly accelerating rate at which OCT images are being acquired in clinical settings. Quantitative parameter estimation allows for the development of objective diagnosis approaches based on correlations between quantified parameter values and the presence of disease. Image classification techniques can be used directly to detect biomarkers of disease, or as a step towards parameter quantification. Finally, image enhancement allows for more accurate expert image interpretation and for improved parameter quantification or classification. The papers contained in this Collection contribute evidence to suggest that quantitative and computational techniques in OCT are crucial to transforming OCT into a tool capable of performing wide-spread, early detection, of a range of diseases.

Finally, the present Collection is still open for submissions on a rolling basis, and with new studies continuing to be submitted, we expect the Collection to serve as a one-stop overview of current research in optical coherence tomography.
